# Underestimation of ammonia‐oxidizing bacteria abundance by amplification bias in *amoA*‐targeted qPCR

**DOI:** 10.1111/1751-7915.12366

**Published:** 2016-05-11

**Authors:** Arnaud Dechesne, Sanin Musovic, Alejandro Palomo, Vaibhav Diwan, Barth F. Smets

**Affiliations:** ^1^ Department of Environmental EngineeringTechnical University of DenmarkMiljoevej, 2800 KgsLyngbyDenmark; ^2^ Present address: Danish Technological InstituteKongsvang Allé 29Aarhus 8000Denmark

## Abstract

Molecular methods to investigate functional groups in microbial communities rely on the specificity and selectivity of the primer set towards the target. Here, using rapid sand filters for drinking water production as model environment, we investigated the consistency of two commonly used quantitative PCR methods to enumerate ammonia‐oxidizing bacteria (AOB): one targeting the phylogenetic gene 16S rRNA and the other, the functional gene *amoA*. Cloning‐sequencing with both primer sets on DNA from two waterworks revealed contrasting images of AOB diversity. The *amoA*‐based approach preferentially recovered sequences belonging to *Nitrosomonas* Cluster 7 over Cluster 6A ones, while the 16S rRNA one yielded more diverse sequences belonging to three AOB clusters, but also a few non‐AOB sequences, suggesting broader, but partly unspecific, primer coverage. This was confirmed by an *in silico* coverage analysis against sequences of AOB (both isolates and high‐quality environmental sequences). The difference in primer coverage significantly impacted the estimation of AOB abundance at the waterworks with high Cluster 6A prevalence, with estimates up to 50‐fold smaller for *amoA* than for 16S rRNA. In contrast, both approaches performed very similarly at waterworks with high Cluster 7 prevalence. Our results highlight that caution is warranted when comparing AOB abundances obtained using different qPCR primer sets.

## Introduction

Investigation of environmental microbial communities by molecular techniques has, over the last decades, demonstrated large advantages, primarily by overriding the limitation associated with microbial cultivation in the laboratory. Although so‐called ‘open format’ methods exist which do not require a priori sequence information from the community of interest, much of the routine monitoring techniques of microbial communities, such as qPCR, are ‘closed‐format’ (i.e. require a priori sequence information) (Zhou *et al*., 2015). These ‘closed‐format’ methods typically depend on the ability of a primer set for unbiased amplification of all (or most) of the target sequences without amplifying non‐target sequences. Primer sets are designed based on the current information from nucleotide databases and require occasional validations as new sequences from the environment are added.

To study bacterial functional groups, primer sets may either target a relevant functional gene or, if the function is performed by one or a few taxa, the phylogenetic 16S rRNA gene. This is the case for the ammonia‐oxidizing bacteria (AOB), which predominantly belong to the beta‐subclass of *Proteobacteria* and carry a functional *amoA*, which codes for a subunit of the ammonia monooxygenase (Norton, 2011). AOB are key players in the first step of nitrification in many natural and engineered systems. To quantify this group, researchers routinely employ qPCR targeting *amoA* or group‐specific 16S rRNA gene sequences (Junier *et al*., 2010). However, as these two approaches are not typically compared, it is unclear whether they are equally good at estimating AOB abundance.

We attempted this comparison, using biomass extracted from biological rapid sand filters (RSF) used for production of potable water from groundwater. One of the key roles of these filters is ammonium removal, which is mediated by ammonia‐oxidizing prokaryotes (AOP) (van der Wielen *et al*., 2009). Occasional failures in ammonium removal call for better understanding and monitoring of AOP communities, which require accurate molecular quantification methods.

Here, we investigate the consistency of two molecular approaches for quantifying AOB: one targeting a phylogenetic gene (16S rRNA) and the other, a functional gene (*amoA*). We tested two commonly applied AOB‐specific primer sets: CTO189a/b/c – RT1r for 16S rRNA (Kowalchuk *et al*., 1997; Hermansson and Lindgren, 2001) and amoA1f ‐ amoA2r (Rotthauwe *et al*., 1997). These primer sets were evaluated for coverage and specificity both in silico and on DNA extracted from biomass from two RSFs. The difference in primer pair selectivity, combined with the compositional differences of the AOB guild across sand filter communities, had a major effect on the quantification outcome.

## Results & Discussion

**Figure 1:**
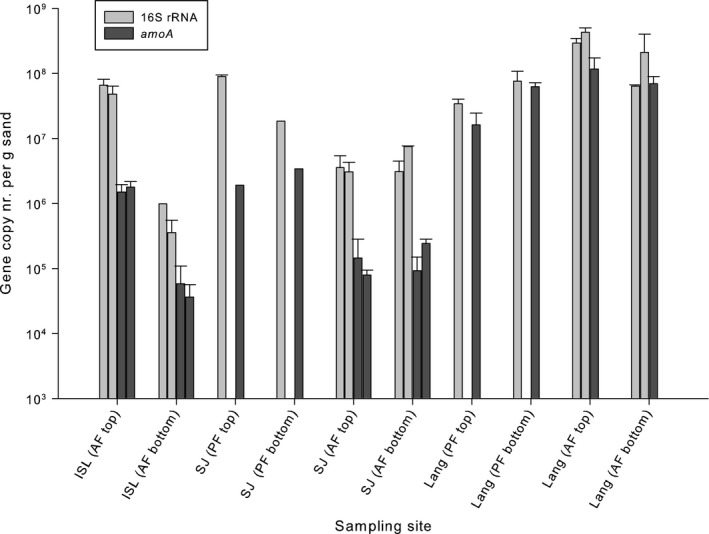
qPCR enumeration of ammonia‐oxidizing bacteria in the top (0–5 cm) and bottom (40–50 cm) layers of rapid sand filters units (pre‐filter ‐PF‐ and after‐filter‐AF) at three Danish waterworks: Islevbro (ISL), Sjælsø (SJ), and Langerød (Lang). The qPCR was performed either by targeting 16S rRNA (primers CTO189a/b/c –RT1r (Kowalchuk *et al*., 1997; Hermansson and Lindgren, 2001)) or *amoA* (primers amoA1f ‐ amoA2r, Rotthauwe *et al*., 1997) genes. When multiple filter units have been sampled at the same waterworks, the data are presented as multiple bars. See supplementary information for details on sampling, DNA extraction, and for qPCR conditions.

Two molecular approaches targeting the phylogenetic 16S rRNA and functional (*amoA*) genes were applied to investigate AOB abundance in pre‐filter and after‐filter units at three Danish waterworks; Islevbro, Sjælsø, and Langerød. The abundance of the AOB 16S rRNA genes ranged from ca. 3 × 10^6^ to ca. 3 × 10^8^ copies/g drained wet sand across RSF units and waterworks (Fig. 1). The abundance estimates were similar between replicate after‐filters within waterworks and indicated some expected within‐plant patterns. Notably, at Sjælsø waterworks, AOB were one‐order of magnitude more abundant in the pre‐ than in the after‐filter units and, in Islevbro after‐filters, a clear two order of magnitude decline of AOB abundance was noted with depth. The enumeration of *amoA* revealed similar AOB distributional trends within and across pre‐ and after‐filters. However, the abundance of *amoA* was consistently lower (ca. 50‐fold) than that of 16S rRNA gene at both Islevbro and Sjælsø waterworks (Fig. S1). This difference becomes even larger considering that the genomes of betaproteobacterial ammonia‐oxidizers typically contain a single ribosomal operon (Stoddard *et al*., 2014) but frequently multiple (1–3) *amo* operons (Norton *et al*., 2002). This inconsistency between the two abundance estimates was not observed at Langerød waterworks.

**Figure 2:**
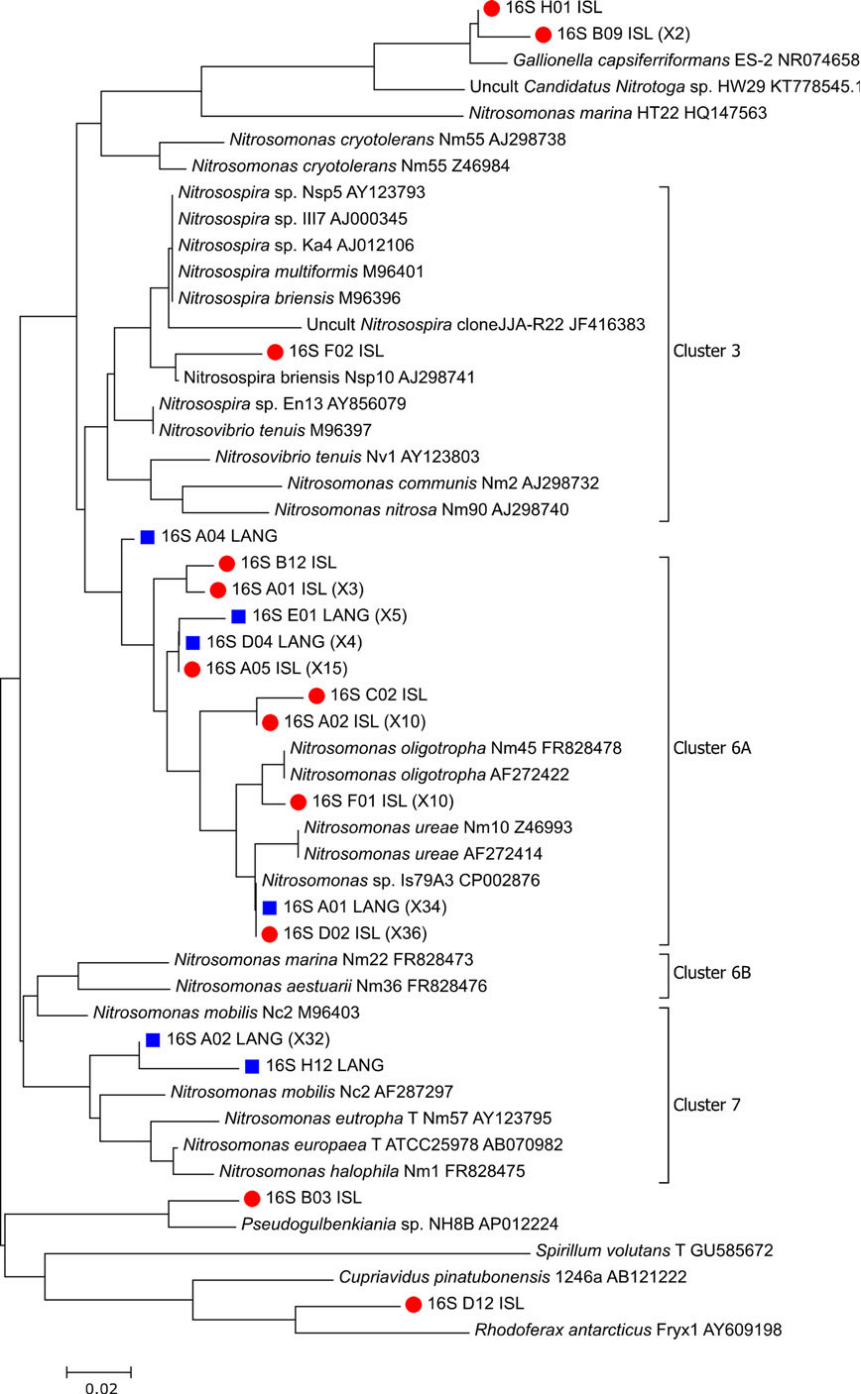
Neighbour‐joining tree of the 16S rRNA gene sequences cloned from after‐filter units at Islevbro (ISL, red circle) and Langerød (LANG, blue square) waterworks along with reference sequences. The cloning was performed with the same primer set as for the qPCR and the sequences have been submitted to DDBJ under accession number LC100143‐100160. The tree was created using the maximum likelihood function of Mega 7 software (Kumar *et al*., 2016) and relies on 41 variable positions. Multiple occurrences of identical sequences are presented in parentheses. The reference strains are presented with the accession numbers after their name.

**Figure 3:**
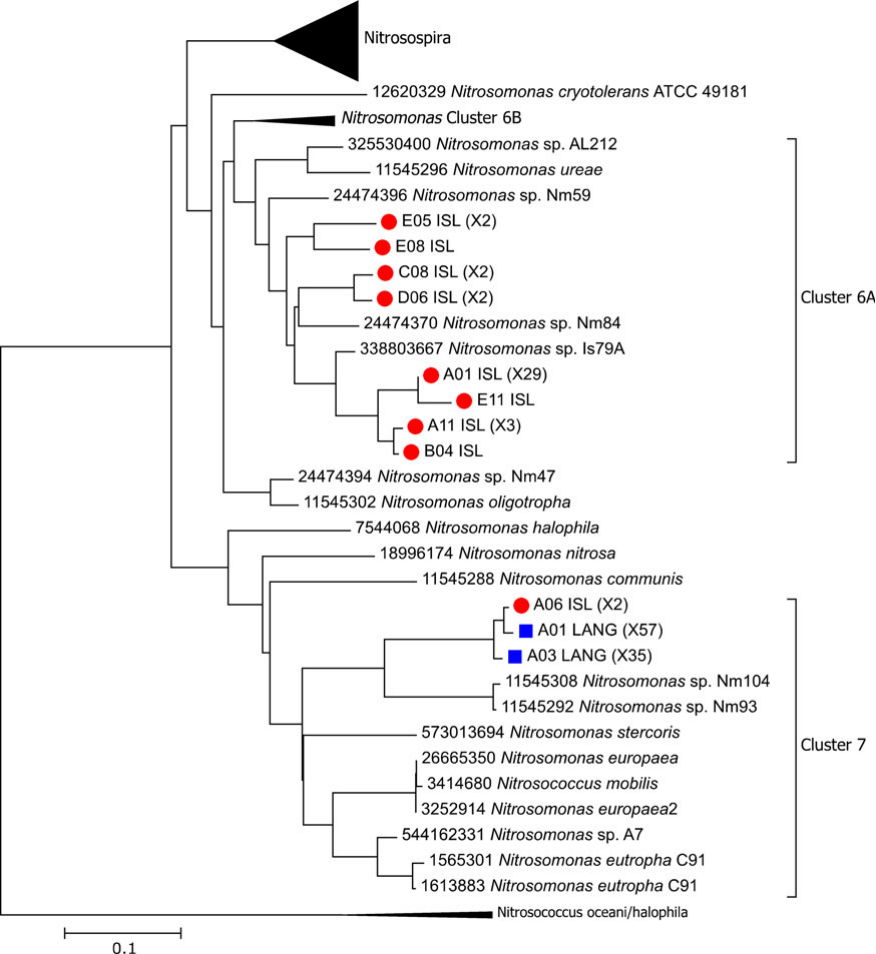
Neighbour‐joining phylogenetic tree inferred from AOB *amoA* gene sequences cloned from after filter‐units at Islevbro (ISL, red circle) and Langerød (LANG, blue square) waterworks and from reference sequences. The cloning was performed with the same primer set as for the qPCR. These sequence data have been submitted to DDBJ under accession number LC142697‐142707. The phylogenetic tree was created using the maximum likelihood function of Mega 7 software (Kumar *et al*., 2016) Multiple occurrences of highly similar sequences (≥ 99% similarity) are presented in parentheses. The reference strains are presented with the sequence identification numbers (GI) numbers in front of their name.

Islevbro and Langerød waterworks, showing, respectively, the largest and smallest differences between the two methods, were selected for cloning‐sequencing analysis in order to examine the selectivity and specificity of the two primer sets towards the AOB. The sequences of the clones recovered from Islevbro and Langerød waterworks (82 and 77 with 16S rRNA genes primers and 43 and 92 for *amoA* primers) were placed into maximum likelihood trees along reference sequences to tentatively classify them (Figs 2 and 3). Phylogenic analysis and classification of the 16S sequences were hindered by the very short length of the amplicon (75 bases). All partial *amoA* sequences and most of the 16S ones were tentatively identified as belonging to Betaproteobacterial AOB (Norton, 2011), but five 16S sequences (6%) were closely related to sequences that did not belong to AOB but to other Beta‐Proteobacteria. (Fig. 2, Table S2). These diverse non‐AOB sequences are thus false positives and indicate that the selectivity of the 16S rRNA primer set is imperfect, which might introduce overestimation of AOB abundance in some communities. We indeed note that these non‐AOB sequences were obtained from one of our clone libraries (Islevbro) but not the other. Imperfect specificity of primer sets targeting the 16S rRNA gene of betaproteobacterial AOB has already been noted (e.g., Mahmood *et al*., 2006). Most clones from Islevbro waterworks were tentatively assigned to *Nitrosomonas* Cluster 6A (*N. oligotropha* lineage), irrespective of the primer set. At Langerød waterworks, in contrast, the identity of the dominant AOB lineage changed depending on the molecular approach: the 16S rRNA‐ based approach identified AOB belonging to Cluster 6A as dominant, while the *amoA*‐based approach only retrieved sequences from the *Nitrosomonas europaea/eutropha* lineage (Cluster 7). While the difference between the diversity retrieved by the two primer sets could also originate from cloning bias (e.g. Palatinszky *et al*., 2011) we further explored the role of amplification bias.

We thus performed an *in silico* coverage analysis of the primer sets against sequences from AOB for which the entire priming region for forward and/or reverse primer was available, collected from RDP (Cole *et al*., 2014) or FunGene databases (Fish *et al*., 2013) for 16S rRNA and *amoA* respectively (number of sequences considered and their distribution into AOB clusters is presented in Table S1). For the 16S rRNA primer set, our analysis revealed a high coverage for both primers across all clusters (Fig. S2), with the forward primer having a slightly worse coverage than the reverse one (zero or one mismatch for more than 91% and 98% target sequences respectively).

The analysis of *amoA* primer set revealed a clear difference between Cluster 6A and the other clusters: the sequences assigned to the former presented, on average, more mismatches to both the forward and the reverse primers (Fig. S2). All Cluster 6A sequences presented at least two, and up to four, mismatches with the primer set while it barely exceeded one, on average, for the other clusters (Fig. S2). The good match between Cluster 7 sequences and the *amoA* primer set is consistent with the high amplification of *N. europaea* sequences reported in a recent qPCR comparative assay (Meinhardt *et al*., 2015). In contrast, the presence of an increasing number of mismatches between a primer and its binding site, in particular two and more mismatches, can significantly reduce the efficiency of PCR amplification (Bru *et al*., 2008). Therefore, we conclude from our *in silico* analysis that the *amoA* primer set would be, on average, less efficient at amplifying Cluster 6 sequences than Cluster 7 ones when both are present. This can explain the lack of detection of Cluster 6A with the *amoA* cloning‐sequencing at Langerød, where both clusters co‐dominated according to the 16S approach (Table S2). However, this co‐dominance of Cluster 6A and 7 did not result in incorrect quantification of AOB by the *amoA* qPCR, which performed similarly as that for the 16S rRNA gene (Fig. 1, Fig. S1). In Islevbro waterworks, cloning‐sequencing with the 16S primer set indicated a strong dominance of Cluster 6A, with Cluster 7 being undetectable. This low abundance of Cluster 7 probably allowed Cluster 6A to retain its high prevalence in the *amoA* clone library, in spite of its low PCR amplification efficiency. This low efficiency, caused by primer‐target mismatches, was, however, apparent in the drastic underestimation of AOB abundance with the *amoA*‐based qPCR at Islevbro (Fig. 1, Fig. S1).

Our *in silico* and experimental results highlight the importance of primer set selection for AOB quantification in complex microbial communities and demonstrate that results can be heavily primer‐ and community‐dependent. Thanks to its consistent coverage across AOB clusters, the primer set targeting the 16S rRNA gene performed better than the *amoA* one which amplifies Cluster 6A at low efficiency. This *amoA* primer set, although commonly used, is thus poorly adapted to analyse communities dominated by this type of AOB. The 16S rRNA gene‐qPCR presents the disadvantage that it can overestimate AOB abundance, although this problem was not detected in one of the waterworks and was modest in the other, with a few percent of non‐target sequences in the clone library. A recent comparison of 16S rRNA and *amoA*‐ based qPCR methods, using primer sets similar, but not identical, to those used here, in activated sludge communities (Baptista *et al*., 2014) revealed that the *amoA*‐based methods yielded abundance estimates very similar to those obtained with FISH, while the 16S rRNA qPCR ones were significantly lower. Their conclusion differs from that of this study and is likely due to compositional differences in the respective communities. Therefore, we recommend, along with (Meinhardt *et al*., 2015) to consider using multiple primer sets when exploring AOB in unknown communities. Inconsistencies between qPCR results would indicate that there is uncertainty in the abundance estimates and that these estimates should be checked with other methods (e.g., qFISH).

## Supporting information


**Data S1.** Material and method.
