# Supplementary material for: Underestimation of ammonia‐oxidizing bacteria abundance by amplification bias in amoA‐targeted qPCR
Source: Microb Biotechnol. 2016 May 11;9(4):519–24. doi: 10.1111/1751-7915.12366 (PMC4919994; doi:10.1111/1751-7915.12366)
Supplement: Supplementary file 1 — Data S1. Material and method. [file MBT2-9-519-s001.docx]

**Supplementary information**

# For ‘Underestimation of ammonia-oxidizing bacteria abundance by amplification bias in *amoA*-targeted qPCR’ by

# Arnaud Dechesne, Sanin Musovic, Alejandro Palomo, Vaibhav Diwan, Barth F. Smets

# Department of Environmental Engineering, Technical University of Denmark, Miljoevej, 2800 Kgs. Lyngby, Denmark

# Material and Method

**Sampling and DNA extraction**

Sand material was collected from the surface (0-10 cm) and the deeper (40-50 cm) layers in rapid sand filters at three Danish waterworks; Islevbro, Sjælsø and Langerød, located 5 to 50 miles from Copenhagen. The sand sampler consisted of a Plexiglas cylinder (1 m height, 5 cm inner diameter) which we pushed deep in, and carefully pulled out from, the rapid sand filter sediment. We recovered intact vertical profiles and cut out the needed layers for further molecular analyses. A pre-filter and two downstream after-filter- units were sampled at Sjælsø and Langerød waterworks. Two after-filter units were sampled at Islevbro waterworks, where the pre-filter consisted of large rocks (Ø, >10 cm) and could not be sampled. The water sampled along with the sand material was drained and half a gram wet weight of sand material was used for genomic DNA extraction using a MP FastDNA™ SPIN Kit (MP Biomedicals LLC., USA) according to manufacturer’s instructions. The quality and quantity of extracted DNA was measured by a NanoDrop™ spectrophotometer (NanoDrop Products, Wilmington, DE).

**PCR-based analyses**

Two PCR-based approaches with either the CTO189a/b/c – RT1r primer set, targeting 16S rRNA gene (Kowalchuk *et al.*, 1997; Hermansson and Lindgren, 2001), or the amoA1f - amoA2r one, targeting *amoA* (Rotthauwe *et al.*, 1997), were evaluated*.* PCRs were run under the following conditions: a 25-μl reaction contained 1 μl of DNA suspension (10 ng DNA), 10 pmol of each primer, 12.5 mM of dNTPs, 1 U *Taq* polymerase and DNA/RNA-free water to 25 µl. The thermal PCR conditions consisted of the initial denaturation step at 95ºC for 5 min., followed by 35 cycles at 94ºC/60s, 60ºC/30s and 72ºC/60s, or 94ºC/60s, 56ºC/30s and 72ºC/30s for 16S rRNA and *amoA* gene primer-sets, respectively. The final DNA extension step was run at 72ºC for 10 min.

Quantification of AOB was made by real-time quantitative PCR (qPCR) analysis with the following conditions: qPCR reaction (25 μl) contained 12.5 µl of 2×iQ SYBR Green Supermix (Bio**-**Rad Laboratories**,** Inc., Hercules, CA), 500 nM of each specific primer (except for CTOfA/B and CTO189C which were mixed in 2:1 molar ratio to a total of 500 nM), 10 ng template DNA and DNA/RNAase free water to 25 µl. For the 16S rRNA gene assay, the template used to construct standard curves was plasmid DNA obtained by cloning a gene fragment amplified from a nitrifying bioreactor with the 27f-1492r primer set (Weisburg *et al.*, 1991). The sequence of this fragment (accession number FJ529920) has tentatively been assigned to *Nitrosomonas*. For the *amoA* qPCR, the standard curve was constructed using an *amoA* amplicon cloned into a plasmid vector. The amplicon was obtained from a PCR with the *amoA* 1f-2r primer set on a DNA extract from *Nitrosomonas europaea.* The qPCRs were run in duplicate on a Chromo4 thermal cycler using Opticon Monitor 3 software (Bio**-**Rad**,** Hercules, CA). The qPCR conditions were identical to the PCR conditions described above, apart from an extension of the number of cycles to 40. A melting curve analysis (gradient 0.2°C/s, range 70-95°C) (Ririe *et al.*, 1997) was performed and no significant presence of primer dimers or unspecific products was observed. The gene abundances were expressed per gram of filter material (wet weight).

**Clone-library construction and analysis**

One clone library was constructed using amplicons from PCR with each primer set. Amplicons were verified on 1 % (wt/vol) agarose gel and purified with a QIAquick PCR purification kit (QIAGEN) prior to ligation into pCR4-TOPO vector (Cloning Kit, Invitrogen) and transformation into competent *Escherichia coli* cells, according to manufacturer’s instructions. Vector-DNA was extracted from clonal cells prior to sequencing (GATC, Germany). The quality of the sequences was evaluated in BioEdit software (Hall, 1999). Ninety-six clones for each clone library were sequenced. For one of the libraries (that constructed with the *amoA* amplicons obtained in Islevbro)*,* about half of the sequenced vectors did not contain any insert, which could either result from a low amplification efficiency or from a low cloning efficiency.

To assign these sequences to AOB clusters, high quality reference sequences (type strains, isolates, or known representatives of clusters without cultivated representative) were collected from Fungene Release 8.2 (Fish *et al.*, 2013), for AOB *amoA*; and from RDP Release 11, Update 4 (Cole *et al.*, 2014), for *Nitrosomonadaceae* 16S rRNA genes. These reference sequences (143 and 167, for *amoA* and 16S rRNA gene, respectively) were used to generate taxonomy outline files, following the AOB classification proposed in (Koops *et al.*, 2006). These files were then used for assigning the sequences of our clones into AOB cluster, using the **classify.seqs function of Mothur v. 1.36 (Schloss *et al.*, 2009) with Wang method and a kmer size of 8. To confirm the assignments, the clone sequences were placed into trees constructed with the reference sequences using** maximum likelihood inference as implemented in Mega 7 (Kumar *et al.*, 2016) and were examined against the NCBI nucleotide database using BLASTn (www.ncbi.nlm.nih.gov/BLAST).

***In silico* coverage analysis**

**Long, high quality AOB 16S rRNA gene and *amoA* sequences were collected from** RDP and FunGene databases. Long sequences were essential to maximize the chances of assigning the sequences to AOB clusters and the chances of including the priming regions of the qPCR assays considered.

The sequences (476 and 5171 for *amoA* and 16S rRNA gene, respectively) were tentatively assigned to AOB clusters using the **classify.seqs function of Mothur as described above. Then, for each sequence, the number of mismatches between the priming regions and the primers were calculated. This was either done using the ProbeMatch function of FunGene for *amoA*, or using a custom-made script in R (R Team, 2015) for the 16S rRNA gene. This script, which uses matching functions available in the package Biostring v2.34.1 (Pages *et al.*), verifies the presence of the priming** region and computes the number of mismatches against the primer. The number of sequences considered and their distribution into AOB clusters is presented in Table S1. Results for clusters represented by less than ten sequences are not reported.

Table S1: Numbers and distribution across AOB clusters of the sequences used for the *in silico* coverage analysis of the *amoA* and 16S rRNA gene primers. No sequence number was reported if the number of retrieved sequences was deemed insufficient for subsequent analysis (<10). The division of AOB into clusters follows (Koops *et al.*, 2006).

|  | **Primer** | | | |
| --- | --- | --- | --- | --- |
| **AOB Cluster** | amoA1F | amoA2R | CTO189fA-B&C | RT1R |
| *Nitrosospira* Cluster 0 | 97 | 97 | 20 | 155 |
| *Nitrosospira* Cluster 1 |  |  | 206 | 2494 |
| *Nitrosospira* Cluster 2 | 17 | 17 | 10 | 56 |
| *Nitrosospira* Cluster 3 | 38 | 16 | 81 | 616 |
| *Nitrosospira* Cluster 4 |  |  | 95 | 129 |
| *Nitrosomonas oligotropha* lineage Cluster 6A | 20 | 20 | 248 | 374 |
| *Nitrosomonas* *marina* lineage Cluster 6B | 16 |  | 47 | 112 |
| *Nistrosomonas europaea / mobilis* lineage Cluster 7 | 24 | 18 | 116 | 214 |
| *Nistrosomonas* *communis* lineage Cluster 8 |  |  | 21 | 46 |
| *Nitrosomonas* sp. Nm143 lineage Cluster 9 | 66 | 66 | 69 | 975 |
| Unassigned sequences | 198 | 198 |  |  |

**Table S2**. Distribution into AOB clusters of the partial 16S rRNA- and *amoA* genes sequences obtained by cloning sequencing rapid sand filters at two Danish waterworks. The assignment was assisted by the sequence.classify function of Mothur. For the *amoA* sequences, the assignment was generally reliable (confidence > 65% for all sequences), but less so for the 16S rRNA gene sequences, due to their short size, although 60% of the sequences were classified with a confidence larger than 75%.

|  | **Waterworks** | | | |
| --- | --- | --- | --- | --- |
| Putative affiliation | **Islevbro** | | **Langerød** | |
|  | **16S rRNA** | ***amoA*** | **16S rRNA** | ***amoA*** |
| *Nitrosomonas oligotropha* lineage (Cluster 6A) | 93% | 95% | 56% | 0 |
| *Nitrosomonas europaea / Nitrosococcus mobilis* lineage (Cluster 7) | 0 | 5% | 43% | 100% |
| *Nitrosospira* Cluster 3 | 1% | 0 | 1% | 0 |
| Other, non Nitrosomonadales, beta-Proteobacteria | 6% | 0 | 0 | 0 |
| **Nr. of clones** | **82** | **43** | **77** | **92** |

**Figure S1: Comparison of *amoA*- and 16S rRNA gene-based qPCR quantification of AOB abundance in the sand material of rapid sand filter at three waterworks*.*** Colors indicate the waterworks (blue: Langerød; red: Islevbro, orange: Sjælsø). Symbols filled black in their upper half indicate samples obtained from the top of the filter, the ones filled black in their bottom half indicate samples obtained from the bottom of the filter. Deviation from the first diagonal (continuous line) indicates difference between the results of the two quantification methods.

**
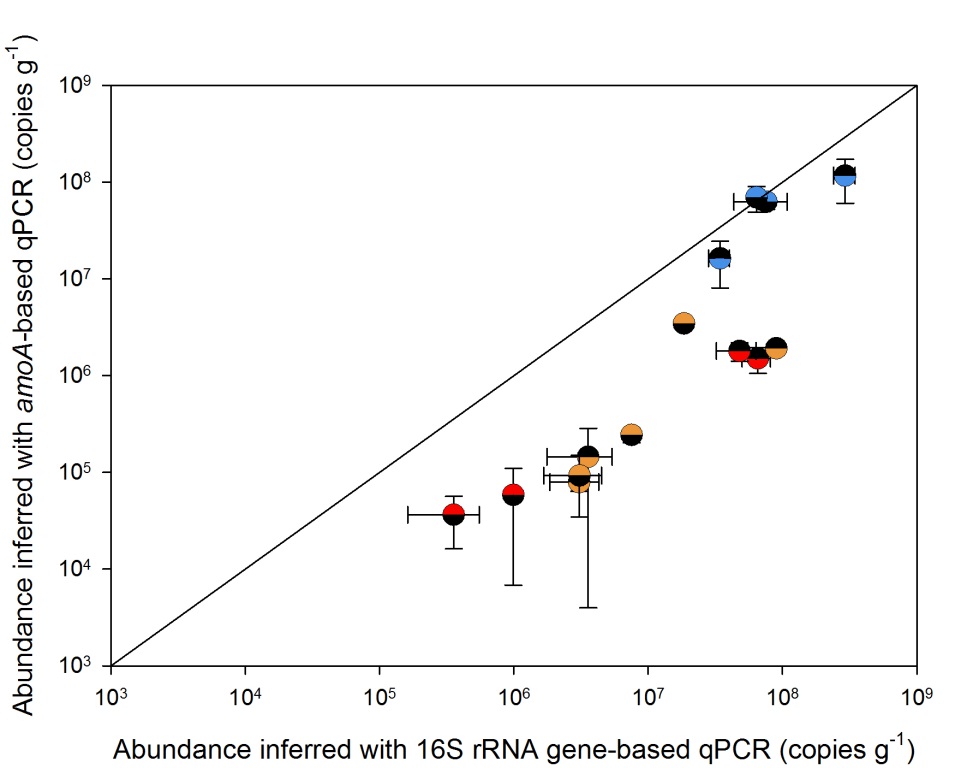
**

**Figure S2: Result of the *in silico* coverage analysis.** Average number of mismatches between target genes (panel A, *amoA*; panel B, 16S rRNA gene) and their respective primer across AOB clusters. Missing clusters are clusters for which sufficient target sequences were not available (see Table S1). Note the difference in y-axis scale between the two panels. The X in panel A indicates unassigned sequences.

**B**

**References**

Cole, J.R., Wang, Q., Fish, J.A., Chai, B., McGarrell, D.M., Sun, Y., et al. (2014) Ribosomal Database Project: data and tools for high throughput rRNA analysis. *Nucleic Acids Res* **42**: D633–42.

Fish, J.A., Chai, B., Wang, Q., Sun, Y., Brown, C.T., Tiedje, J.M., and Cole, J.R. (2013) FunGene: the functional gene pipeline and repository. *Front Microbiol* **4.**:

Hall, T. (1999) BioEdit: a user-friendly biological sequence alignment editor and analysis program for Windows 95/98/NT. *Nucleic Acids Symp Ser* **41**: 95–98.

Hermansson, A. and Lindgren, P.E. (2001) Quantification of ammonia-oxidizing bacteria in arable soil by real-time PCR. *Appl Environ Microbiol* **67**: 972–6.

Koops, H., Purkhold, U., Pommerening-Röser, A., Timmermann, G., and Wagner, M. (2006) The Lithoautotrophic Ammonia-Oxidizing Bacteria. In, *The Prokaryotes*. Springer New York, New York, NY, pp. 778–811.

Kowalchuk, G.A., Stephen, J.R., De Boer, W., Prosser, J.I., Embley, T.M., and Woldendorp, J.W. (1997) Analysis of ammonia-oxidizing bacteria of the beta subdivision of the class *Proteobacteria* in coastal sand dunes by denaturing gradient gel electrophoresis and sequencing of PCR-amplified 16S ribosomal DNA fragments. *Appl Environ Microbiol* **63**: 1489–97.

Kumar, S., Stecher, G., and Tamura, K. (2016) MEGA7: Molecular Evolutionary Genetics Analysis version 7.0 for bigger datasets. *Mol Biol Evol* msw054.

Pages, H., Aboyoun, P., Gentleman, R., and DebRoy, S. Biostrings: String objects representing biological sequences, and matching algorithms. *2015*.

Ririe, K.M., Rasmussen, R.P., and Wittwer, C.T. (1997) Product Differentiation by Analysis of DNA Melting Curves during the Polymerase Chain Reaction. *Anal Biochem* **245**: 154–160.

Rotthauwe, J.H., Witzel, K.P., and Liesack, W. (1997) The ammonia monooxygenase structural gene *amoA* as a functional marker: molecular fine-scale analysis of natural ammonia-oxidizing populations. *Appl Environ Microbiol* **63**: 4704–12.

Schloss, P.D., Westcott, S.L., Ryabin, T., Hall, J.R., Hartmann, M., Hollister, E.B., et al. (2009) Introducing mothur: open-source, platform-independent, community-supported software for describing and comparing microbial communities. *Appl Environ Microbiol* **75**: 7537–41.

Team, R.D.C. (2015) R: a language and environment for statistical computing.

Weisburg, W.G., Barns, S.M., Pelletier, D.A., and Lane, D.J. (1991) 16S ribosomal DNA amplification for phylogenetic study. *J Bacteriol* **173**: 697–703.
